# Integration of an EEG biomarker with a clinician's ADHD evaluation

**DOI:** 10.1002/brb3.330

**Published:** 2015-03-05

**Authors:** Steven M Snyder, Thomas A Rugino, Mady Hornig, Mark A Stein

**Affiliations:** 1 Department of Research and Development, NEBA HealthBoulder, Colorado; 2 Department of Psychiatry and Health Behavior, Georgia Regents UniversityAugusta, Georgia; 3 Children's Specialized HospitalToms River, New Jersey; 4 Robert Wood Johnson School of MedicinePiscataway, New Jersey; 5 Department of Epidemiology, Mailman School of Public Health, Columbia UniversityNew York, New York; 6 Department of Psychiatry and Behavioral Science, and Pediatrics, University of WashingtonSeattle, Washington; 7 Department of Psychiatry and Behavioral Medicine, Seattle Children's HospitalSeattle, Washington

**Keywords:** Attention deficit hyperactivity disorder, biomarkers, comorbidity, electroencephalography, multidisciplinary, sensitivity, specificity

## Abstract

**Background:**

This study is the first to evaluate an assessment aid for attention-deficit/hyperactivity disorder (ADHD) according to both Class-I evidence standards of American Academy of Neurology and De Novo requirements of US Food and Drug Administration. The assessment aid involves a method to integrate an electroencephalographic (EEG) biomarker, theta/beta ratio (TBR), with a clinician's ADHD evaluation. The integration method is intended as a step to help improve certainty with criterion E (i.e., whether symptoms are better explained by another condition).

**Methods:**

To evaluate the assessment aid, investigators conducted a prospective, triple-blinded, 13-site, clinical cohort study. Comprehensive clinical evaluation data were obtained from 275 children and adolescents presenting with attentional and behavioral concerns. A qualified clinician at each site performed differential diagnosis. EEG was collected by separate teams. The reference standard was consensus diagnosis by an independent, multidisciplinary team (psychiatrist, psychologist, and neurodevelopmental pediatrician), which is well-suited to evaluate criterion E in a complex clinical population.

**Results:**

Of 209 patients meeting ADHD criteria per a site clinician's judgment, 93 were separately found by the multidisciplinary team to be less likely to meet criterion E, implying possible overdiagnosis by clinicians in 34% of the total clinical sample (*93/275*). Of those 93, 91% were also identified by EEG, showing a relatively lower TBR (*85/93*). Further, the integration method was in 97% agreement with the multidisciplinary team in the resolution of a clinician's uncertain cases (*35/36*). TBR showed statistical power specific to supporting certainty of criterion E per the multidisciplinary team (*Cohen's d, 1.53*). Patients with relatively lower TBR were more likely to have other conditions that could affect criterion E certainty (*10 significant results; P ≤ 0.05*). Integration of this information with a clinician's ADHD evaluation could help improve diagnostic accuracy from 61% to 88%.

**Conclusions:**

The EEG-based assessment aid may help improve accuracy of ADHD diagnosis by supporting greater criterion E certainty.

## Introduction

Attention-deficit/hyperactivity disorder (ADHD) is characterized by the presence of developmentally inappropriate attentional and behavioral symptoms according to the criteria outlined in the DSM-IV-TR and DSM-V (Diagnostic and Statistical Manual of Mental Disorders) (APA 2000, 2013). The criteria also require that the symptoms are not better explained by another disorder (i.e., criterion E). This complicates ADHD evaluation because ADHD-like symptoms are known to be present in other psychiatric disorders as well as in medical and neurological conditions (Zametkin and Ernst 1999; Mirsky and Duncan 2001; Daley 2004).

To address the challenges of ADHD diagnosis, professional guidelines recommend a comprehensive evaluation of available clinical information including that from assessment aids (AACAP 2007; AAP 2011). Although ADHD is viewed as a neurodevelopmental disorder, current recommendations for assessment aids do not include biomarkers (biologically derived indicators of ADHD). A number of potential biomarkers are being investigated in neurophysiology, neuroimaging, neurochemistry, and genetics (Sowell et al. 2003; Bush et al. 2005; Cortese 2012). Many biomarker findings have been consistent in associating frontal cortical abnormalities with an ADHD diagnosis (Barry et al. 2003; Dickstein et al. 2006; Monastra 2008; Cortese 2012).

For biomarkers to be of clinical utility, integration into the clinic may require a strategy other than noting an association with ADHD (Bush 2008). One possible integration method may include conducting a clinician's regular ADHD evaluation in series with a biomarker test. In such a series, a decision-algorithm could be established such that the power of a clinician's case-finding sensitivity could be combined with the potential power of a biomarker's specificity (similar to the HIV testing strategy, which includes an algorithm to combine two tests in series to enhance overall accuracy) (Chou et al. 2005). Longstanding issues affecting the current diagnostic process include the subjectivity of ADHD symptoms, modest agreement between parents and teachers, as well as overlap of attentional and behavioral symptoms with other disorders (Cantwell 1996; Zametkin and Ernst 1999; APA 2013). Therefore, a biomarker that could result in a more homogenous or restricted phenotype of ADHD as the primary diagnosis (the initial focus of treatment/management to address attentional, behavioral, and/or other concerns) would be clinically useful. Eventually, this may lead to subgroups of individuals with ADHD symptoms that have a more specific etiology, course, or response to treatment than ADHD based on descriptive features or symptoms alone (Cantwell and Baker 1987). Such an approach is consistent with the recent Research Domain Criteria project of the US National Institute of Mental Health, with smaller, more fine-grained domains or units of behavior or function that have been shown to be associated with disruptions of neural circuitry (Insel et al. 2010).

Therefore, we proposed a method to integrate an electroencephalographic (EEG) biomarker (theta/beta ratio, TBR) in series with a clinician's regular ADHD evaluation for the purpose of improving criterion E certainty to assist in determining whether ADHD is the primary diagnosis. We developed an EEG integration method intended to distinguish pediatric subgroups that vary in their likelihood of having conditions that may account for ADHD symptoms or otherwise impact ADHD diagnosis and management. Clinically, the capacity to identify a subgroup that is less likely to have such complicating conditions (i.e., more likely to meet criterion E) would enhance clinician confidence in rendering a primary diagnosis of ADHD. Likewise, the ability to identify a subgroup that is more likely to have such conditions (i.e., less likely to meet criterion E) would provide support for clinicians in suggesting further examination of the child prior to making a diagnostic conclusion of ADHD and/or proceeding with treatment planning, increasing the efficiency of the diagnostic process.

To validate the EEG integration method, we examined concordance with a reference standard defined as consensus best estimate diagnosis of an independent multidisciplinary clinical team (neurodevelopmental pediatrician, clinical psychologist, and child/adolescent psychiatrist). Previous clinical findings have shown that when a multidisciplinary model is applied, a significant number of patients presenting with ADHD-like concerns may be determined as having other primary diagnoses (Pearl et al. 2001, 2015). Therefore, a multidisciplinary model is well-suited to evaluate a biomarker integration method intended to improve diagnostic clarity by supporting greater certainty with criterion E. As such, the current study evaluated whether the proposed EEG integration method could help to improve diagnostic accuracy of the clinician's ADHD evaluation. The current report provides an expanded view of study results that had been presented in regulatory documentation (FDA 2013), and provides further analyses and results that address related questions of the field, as guided by the journal peer review.

## Methods

### Outline of integration method

The integration method is applied to children and adolescents who present to a clinic with attentional and behavioral concerns. The clinician first performs their regular ADHD evaluation. The EEG biomarker (TBR) is applied as a next step intended to improve certainty with criterion E. Relatively lower TBR is used to note cases more likely to have complicating conditions such as medical mimics (i.e., less likely to meet criterion E). The clinician ultimately would resolve those cases with their own judgment, performing further testing for other conditions as they see fit on a patient-by-patient basis.

To function, the integration method requires input from a clinician's ADHD evaluation and from a standardized TBR measurement, and yields recommendations based on a predefined decision-algorithm (Table 1). After the clinician performs an initial evaluation and determines positive/uncertain/negative for ADHD, the biomarker outcome is used to separate patients with clinician designations of “uncertain” and “positive” (ADHD) into EEG-based subgroups with recommendations regarding: (1) ADHD confirmation, or (2) criterion E certainty (with a suggestion for resolution by further clinical testing). The biomarker is not applied to patients with a clinician “negative” designation; in other words no ADHD diagnosis is possible without the clinician's determination of ADHD criteria.

**Table 1:** Outline of integration method

	Clinician's ADHD Evaluation	
	ADHD	Uncertain
**EEG: Theta/Beta Ratio**		
High	ADHD confirmed	ADHD more likely to be confirmed^1^
Low to Moderate	Less likely to meet criterion E^2^	Less likely to meet criterion E^2^

1 Recommend resolution by further clinical testing for ADHD.

2 Recommend resolution by further clinical testing for other conditions. (Note: By the integration method, ADHD negative cases are always solely determined by the clinician.)

### Overview of investigation

The validation study met Class I evidence requirements for ADHD assessment aids per the American Academy of Neurology and the American Clinical Neurophysiology Society (Nuwer 1997). The study was also the first in which validation of an ADHD assessment aid met De Novo evidence requirements per the US Food and Drug Administration (FDA 2013). To evaluate the predefined recommendations of the proposed integration method, investigators conducted a prospective, triple-blinded, 13-site, clinical cohort study. To minimize bias, independent third-party agencies maintained regulatory standard protocols including blinding, monitoring, and database compilation/locking. Investigators collected comprehensive clinical evaluation data from 275 children and adolescents presenting with attentional and behavioral concerns. At each site, a qualified clinician (psychiatrist, psychologist, pediatrician, or physician qualified to assess psychiatric disorders and experienced in diagnosing ADHD) performed differential diagnosis and designated the primary diagnosis (the initial focus of treatment/management to address attentional, behavioral, and/or other concerns). EEG was collected by separate teams. After blind-break, analyses were performed to evaluate and compare the integration method (clinician's regular evaluation plus EEG) and the clinician alone, in terms of criterion E certainty and diagnostic accuracy. The reference standard was the consensus best estimate diagnosis of a multidisciplinary team (child/adolescent psychiatrist, clinical psychologist, and neurodevelopmental pediatrician) that had independently reviewed the clinical data blinded to EEG.

### Ethical considerations

Institutional review boards for each site approved the protocol. The study including informed consent was conducted in accord with US Food and Drug Administration regulations, guidelines for Good Clinical Practice, and the Declaration of Helsinki. Diagnostic methods were modeled according to the American Academy of Child and Adolescent Psychiatry (AACAP) practice parameter for ADHD (AACAP 2007). The study included a follow-up visit at which the site clinician could share results of their clinical evaluation with subject and family.

### Subject population and sample size

The recruited sample was intended to be representative of patients for whom the next step after clinical presentation would be full ADHD evaluation, including application of assessment tools such as the proposed biomarker integration method. Recruits were 364 children and adolescents ages 6.00–17.99 years consecutively presenting with attentional and behavior concerns to 13 geographically distinct clinics (5 psychiatric, 3 psychological, 5 physician/pediatric) in United States from December 11th, 2007 to June 16th, 2008. For inclusion in the study, subjects needed to be willing to stop: (1) psychiatric medications, (2) prescription or nonprescription medications with psychoactive properties, and (3) any medication that might affect EEG. Exclusion criteria were: (1) previous diagnosis of mental retardation or intelligence quotient (IQ) <70; (2) history of seizure disorder, EEG abnormalities, or anticonvulsant use for seizure control; (3) metal plate or device in the head; (4) suicidal ideation or gesture and/or homicidal ideation or gesture; and (5) known serious medical problems (cardiovascular, hematological, or chronic respiratory problems).

Of the 364 recruits, three were excluded due to IQ<70, 1 was excluded due to suicidal ideation, 1 was removed by the investigator to restart an asthma medication, and 1 was withdrawn by the parent to restart an antipsychotic medication. Sixteen recruits did not complete the study, 35 had incomplete EEG recordings related to external issues with computer power supply and impedance meter, and 32 did not receive a complete EEG recording addressed below in Results: Missing Data Analysis. As such, there were 275 subjects who met protocol criteria, completed the study, and had complete EEG recordings. All 275 subjects were included in analysis of diagnostic accuracy.

Sample demographics are as follows (*n* = 275): (1) adolescents 27%; children 73%; (2) female 36%; male 64%; (3) African American 17%; Asian American 1%; Caucasian 73%; Hispanic/Latino 4%; Native American 2%; Other 4%; (4) upper/upper-middle class 16%; lower middle class 33%; working class 33%; lower class 18%. Clinical characteristics by mean (standard deviation) include: (1) age in years 10.1 (2.9); (2) Children's Global Assessment Scale (CGAS) 56 (9); (3) Clinical Global Impression-Severity subscale (CGI-S) 3.7 (1.0); (4) Investigator ADHD-IV Rating Scales (ADHD-IV RS) percentile: Inattention 94 (13); Hyperactivity 86 (21); Total 93 (12); (5) Teacher ADHD-IV RS percentile: Inattention 74 (24); Hyperactivity 69 (28); Total 74 (23); (6) Wechsler Abbreviated Scale for Intelligence-long version (WASI), estimated full scale IQ 102 (14); (7) Wide Range Achievement Test-4 (WRAT-4) Standard Score: Spelling 99 (15); Math 98 (15); Reading 100 (14); Comprehension 98 (16); Reading Composite 98 (15). Socioeconomic status was estimated by an academic class model (Thompson and Hickey 2005) using educational attainment and occupational status for main and secondary providers.

### Medication control

Some of the consecutively presenting participants had prior psychiatric diagnoses with treatment management, and were seeking a second evaluation. Therefore, recruited subjects on medications required a washout plan determined and monitored by the site investigator. Washout depended on the clinician's judgment; recommendations in the protocol included: (1) psychostimulants, at least 1 week prior to study entry, (2) other psychiatric medications, at least 2 weeks prior to study entry, and (3) fluoxetine, 28 days prior to study entry. Of the 275 subjects included in final analysis, 13 (5%) required medication washout prior to study entry. No adverse effects related to medication withdrawal were reported over the course of the study.

### Clinician's ADHD evaluation

All data collection was conducted over visits on four different days to minimize subject fatigue. Investigators collected clinical evaluation data from patients by administering: (1) Physical examinations, including vital signs, vision/hearing screens, and medical/neurological/medication histories, (2) Clinician interviews, with initial impressions and reference to DSM-IV-TR criteria (APA 2000), (3) Kiddie-Schedule of Affective Disorders and Schizophrenia–Present and Lifetime Version (K-SADS-PL) and Supplements with interviewer notes, (4) CGAS, (5) CGI-S, (6) ADHD-IV RS completed by investigator with parent informant, (7) ADHD-IV RS completed by 1–2 teachers, (8) WASI, (9) WRAT-4, (10) Questionnaires on socioeconomic status, education and family histories, and (11) any further testing if deemed necessary by the clinician on a patient-by-patient basis (e.g., for children suspected of central auditory processing or autism spectrum disorders). Informants included child, parent(s), and 1–2 teachers (depending on availability).

Reviewing the above-listed data, a qualified clinician (psychiatrist, psychologist, pediatrician, or physician qualified to assess psychiatric disorders and experienced in diagnosing ADHD) at each site performed and recorded differential diagnosis for ADHD and other disorders and conditions. Extra focus was recommended for disorders and conditions that could account for ADHD-like symptoms. Diagnostic evaluations were guided by the DSM-IV-TR criteria and AACAP practice parameter.

In addition, the clinician summarized their own impressions and judgments for: (1) primary diagnosis, (2) certainty regarding presence of ADHD, and (3) certainty regarding primary diagnosis. Impressions were based on the clinician's whole evaluation of the patient, in which the clinician could describe suspicions beyond DSM-IV-TR criteria. Certainty included addressing whether the diagnosis should be a clinical concern based on symptoms, impairment, and overall coherence of the clinical profile.

To implement these diagnostic results as part of the study analysis of the proposed EEG integration method, the clinician's diagnostic conclusions were summarized as “positive”, “negative” or “uncertain” for ADHD. ADHD was listed as “positive” for the analysis if the clinician's primary diagnosis was ADHD (combined, hyperactive/impulsive, or inattentive subtypes) with definite certainty. ADHD was considered as “negative” for the analysis if the clinician's primary diagnosis was for another condition, and the clinician listed ADHD as absent or secondary. ADHD was considered as “uncertain” in the remaining cases that involved different reported levels of uncertainty.

### EEG collection and analysis

At each clinical site on two different subject visits (weeks apart: mean 2.5; standard deviation 1.7; range 0.1–11.9; median 2.1), a data collection technician or clinician (who each had received training standardized to the collection system) recorded digital EEG data (Compact EEG-Investigational; NEBA Health, Augusta, GA). The standardized EEG collection protocol included recommendations for the subject regarding optimal EEG collection such as for sleep prior to the day of recording. The planned EEG analysis required a single recording electrode (CZ) placed in accordance with the International 10–20 system using an electrode headband (ground electrode near FZ; linked ears reference). Electro-oculography was used to monitor eye blinks and movement. Electrode impedances were to be adjusted (≤10 kΩ). EEG specifications included 256 Hz sampling rate and corner frequencies at 0.5 and 36 Hz. EEG data were collected for ten minutes while patients were seated in a straight-back chair with eyes open and fixed with attention to a point at eye level on the wall.

To limit noise in the EEG data, artifacting and processing technicians at a central EEG processing center screened out epochs containing the most artifact (using a 50 *μ*V threshold followed by visual recognition per standardized methods; NEBA Health). Analysis of EEG data required at least 15 epochs (30 sec) of data with minimal to no artifact. Analysis was conducted using Fast Fourier Transform analysis (frequency resolution: 0.5 Hz) with data resampled at 128 Hz. TBR was examined using a standardized EEG collection and processing system (NEBA Health, Augusta, GA, USA).

Prior to blind-break in the current study, an independent development data set collected in a previous study (Snyder et al. 2008) was used to establish a new set of TBR cutoffs redesigned specifically to support the approach of the proposed integration model, as well as standardized to current system hardware/software parameters. The cutoffs were established for ages 6.00–11.99 and 12.00–17.99 years with the objective of parsing patients with attentional and behavioral concerns into test-result subgroups with clinically meaningful differences relevant to ADHD diagnostic certainty, as well as to the need for more extended medical and neuropsychiatric evaluation, in particular to address criterion E certainty. In the integration method, EEG result categories were labeled for reference as “low”, “moderate”, or “high” for TBR level.

Because nondisclosure of the TBR cutoff values has been permitted, an outline of the construction method has been provided, as follows. TBR cutoff values were determined in a multi-step process that included estimation of measurement error, consideration of risks of false results within context of the intended use, and analyses of population distributions of TBR (for children and for adolescents separately). First, an interval width for a zone now labeled “moderate TBR” was developed to account for measurement error due to variability from artifacting, data collection, and data processing that might not be fully addressed by standardization and calibration, as determined by analyses of the development data, as well as by engineering evaluation of systems and methods. Second, consideration of risk of false results in the context of use according to expert consultation and other applicable resources (e.g., examination of between-site variation in the previous study) supported development of an integration method that could reduce risk by favoring specificity of TBR in cutoff development (made feasible in part by relying more on the clinician's evaluation to support sensitivity in the integration method). Third, development data sets from children and adolescents, who all had ADHD-like symptoms but only a portion had received a clinician's ADHD diagnosis, were used to produce age-based distributions of: (1) subjects with ADHD-like symptoms due to ADHD, and (2) subjects with ADHD-like symptoms better accounted for by other conditions. Fourth, as informed by distribution analyses, the measurement error-based interval (“moderate TBR”) was positioned within the ADHD/non-ADHD overlap to favor specificity in a manner considered optimal for lowering risk per the integration method. As a view of the ultimate distribution of the development data (with ADHD presence determined per the reference standard of the previous study), 75% of subjects with a clinician's ADHD diagnosis were in the high TBR range (above the moderate interval), 6% were in the moderate range, and 19% were in the low range (below the moderate interval). In addition, 13% of those who had ADHD-like symptoms better accounted for by other conditions were in the high TBR range, 11% in the moderate, and 76% in the low. Finally, it should be noted that between-site variation in application of the previous study's reference standard – particularly with respect to differential diagnosis – supported the insight that many “low to moderate TBR” cases receiving an ADHD diagnosis per individual clinicians might have benefited from further rigorous consideration of criterion E, as addressed now in the design of the integration method.

After blind-break in the current study, the integration method was used to parse subjects into test-result subgroups based on the clinician's ADHD diagnostic result and the EEG result, which in turn designated predefined recommendations regarding criterion E certainty and primary diagnosis (see Table 1 and Methods: Outline of Integration Method).

### Reference standard for validation

To evaluate the predefined recommendations of the integration method, a reference standard was produced in which a multidisciplinary team independently determined consensus best estimate diagnosis by separate, off-site review of the de-identified patient files. The multidisciplinary team comprised a clinical psychologist, a neurodevelopmental pediatrician, and a child/adolescent psychiatrist, who worked together in a multidisciplinary clinic for attention-related psychiatric and medical conditions (HALP Clinic, Chicago, IL, USA). Team qualifications included over 25 years of ADHD specialization by the team leader, and regular application of multidisciplinary model in research and clinical practice. Patient files included all clinical evaluation data, except for blinding to EEG and site clinician's diagnoses (as well as parent rating scales, to avoid the bias of repeating information already covered in K-SADS-PL by the same informant). Team members independently reviewed patient files and recorded initial impressions. Then, the multidisciplinary team met and determined consensus best estimate diagnosis for ADHD, as well as differential diagnosis for other disorders and conditions, guided by DSM-IV-TR criteria. Consensus was reached per standard practice of the multidisciplinary clinic, which included a team discussion of each patient with contributions from each member based on perspectives and insights from their specializations. With the understanding gained from the multidisciplinary discussion, each member reconsidered prior opinions and formed a team consensus diagnosis. The team also described recommendations for further testing, in particular to address criterion E certainty. The team summarized diagnostic results by referring to their DSM-IV-TR guided diagnoses and then applying their impressions and clinical judgment regarding: (1) primary diagnosis, (2) certainty regarding presence of ADHD, and 3) certainty regarding primary diagnosis.

### Statistical analysis

We hypothesized that the proposed method to integrate an EEG biomarker in series with a clinician's ADHD evaluation would improve diagnostic accuracy. To test the hypothesis, diagnostic accuracies associated with “clinician's regular evaluation plus EEG” were compared with estimates of those achieved by “clinician alone”. Because one outcome of the integration method is “less likely to meet criterion E” leading to a “recommendation for further testing for other conditions”, this outcome was evaluated in the study as follows. During the comprehensive clinical data collection, clinicians were free to use judgment to apply any further testing on a patient-by-patient basis. After the clinician's initial evaluation and diagnosis were completed, the integration method designated potential cases less likely to meet criterion E in which even more “further testing” may have been needed than the clinician initially provided. To evaluate both clinician judgment and EEG-based feedback regarding further testing and criterion E certainty, an independent multidisciplinary team reviewed the clinician's collected data and determined whether even more “further testing” was indeed needed in particular regarding conditions that could impact the primary diagnosis. To evaluate diagnostic accuracy results in terms of criterion E certainty as produced by the integration method, sensitivity, specificity, positive predictive value, negative predictive value, and overall accuracy were analyzed with reference to: (1) best estimate diagnosis results of multidisciplinary team, (2) presence of complications from predefined study list determined per patient files and multidisciplinary team, (3) suggestion by multidisciplinary team for further testing for conditions on predefined study list, and (4) suggestion by multidisciplinary team that further information may be needed related to differential diagnosis. Confidence intervals (95% CI) were reported with all accuracy results (Agresti and Coull 1998).

To examine clinical differences between test-result subgroups of the integration method, χ^2^ analysis was used (significance level 0.05). Because comparisons between subgroups were complementary and few in number, a multiple testing correction was performed to control for false discovery rate (Benjamini and Hochberg 1995), which shifted the significance level to 0.042. As further support, this selected correction is recommended to be applied to results that support further investigation (Brown and Russell 1997), and the current study's group difference results are intended to support the integration method's outcome of “*recommend resolution by further clinical testing for other conditions*” (see Table 1: footnotes and Methods: Outline of Integration Method).

To evaluate reliability, intraclass correlation coefficient (ICC) analysis was performed on TBR repeated measures, collected on two different visits (*n* = 198). TBR measures were ordered by 1st and 2nd recordings, which could produce an ordering effect because of increased familiarity of subject with EEG at 2nd recording. Therefore, the ICC model chosen was two-way, random, single-measure, consistency: ICC(C,1). In the current report to address concerns of the journal peer review regarding TBR effect size and age effects, further analyses were included applying Cohen's *d* calculations, one-way ANOVA (significance level, 0.05), and linear fits as per previous studies (Arns et al. 2013; Liechti et al. 2013; Loo et al. 2013).

### Triple-blinding and other controls of bias

To minimize bias, independent third-party agencies maintained regulatory standard protocols for blinding, monitoring, data management, site queries, and database compilation and locking. All data were collected with triple-blinding between three sources: (1) site: clinical data collection and clinician's diagnoses, (2) EEG: site collection and off-site processing, (3) multidisciplinary team: diagnoses. Prior to blind-break, clinical data, EEG, and diagnostic results were locked in databases by third-party agencies independent of study sponsor. After blind-break, all analyses were performed on data from the locked and controlled databases per predefined statistical analysis plans or regulatory agency guidance. Study data were submitted to regulatory agencies which repeated analyses and confirmed results.

## Results

### Classification results

In the proposed integration method, the clinician first performs their regular ADHD evaluation. EEG is applied as a next step intended to improve certainty with criterion E. Relatively lower TBR is used to note cases less likely to meet criterion E. Relatively higher TBR is used to confirm ADHD (Table 1).

To validate, the reference standard was consensus diagnosis by a multidisciplinary team, which is well-suited to evaluate criterion E in a complex clinical population in which all subjects presented with attentional and behavioral concerns but not all had ADHD. The diagnostic evaluation of the multidisciplinary team included collection of information and conclusions to address the outcomes of the integration model. For patients meeting ADHD criteria per a site clinician's judgment, integration method outcomes were: (1) ADHD confirmed, or (2) Less likely to meet criterion E. Table 2a presents classification results for these patients.

**Table 2:** (a) Classification results support that the integration method (Clinician + EEG) can help to resolve certainty of criterion E in Clinician's ADHD cases. (b) Classification results support that the integration method (Clinician + EEG) can help to resolve Clinician's uncertain cases

	Multidisciplinary Team	
	ADHD confirmed	Less likely to meet criterion E
(a)		
**Clinician**		
ADHD	116	93
**Clinician + EEG**		
*Clinician*: ADHD		
*EEG (higher TBR)*: ADHD confirmed	95	8
*EEG (lower TBR)*: Less likely to meet criterion E	21	85

	Multidisciplinary Team	
	ADHD more likely to be confirmed	Less likely to meet criterion E
(b)		
**Clinician**		
Uncertain	11	25
**Clinician + EEG**		
*Clinician*: Uncertain		
*EEG (higher TBR)*: ADHD more likely to be confirmed	11	1
*EEG (lower TBR)*: Less likely to meet criterion E	0	24

Note: By the integration method, ADHD negative cases are the same for Clinician and for Clinician + EEG (see Table 1: footnote), and there were 3 false positives and 27 true negatives, as determined per results of the Multidisciplinary Team.

Per results in Table 2a, of the 209 patients meeting ADHD criteria according to an individual clinician's judgment, 93 were separately found by the multidisciplinary team to be less likely to meet criterion E, implying possible overdiagnosis by individual clinicians in 34% of the total clinical sample (*93/275*). Of those 93, 91% were also identified by EEG, showing a relatively lower TBR (*85/93*).

Of the 116 ADHD cases confirmed by the multidisciplinary team, 21 were identified by the integration method as less likely to meet criterion E, implying an unnecessary prompt for further clinical testing by the integration method in 8% of the total clinical sample (*21/275*).

Finally, it should be noted that by the integration method, ADHD-negative cases are the same by definition for clinician and for “clinician + EEG” (Table 1: footnote), and there were 3 false positives and 27 true negatives, as determined per results of the multidisciplinary team (Table 2: footnote). By the above terms and classification results as presented in Table 2a, overall accuracy for the clinician's regular evaluation was 60% [*(116 + 27)/(116 + 93 + 3 + 27)*]. And, overall accuracy for the integration method was 87% [*(95 + 85 + 27)/(95 + 8 + 21 + 85 + 3 + 27)*].

In addition to addressing certainty of criterion E in patients meeting ADHD criteria per a clinician's judgment, the integration method also provides recommendations intended to help resolve a clinician's uncertain cases (who all had presented with attentional and behavioral concerns). Relatively lower TBR is used to note cases less likely to meet criterion E (Table 1). And, relatively higher TBR is used to note cases more likely to have ADHD confirmed. The diagnostic evaluation of the multidisciplinary team included collection of information and conclusions to address these outcomes. Table 2b presents classification results for these patients.

Per results in Table 2b, the integration method was in 97% agreement with the multidisciplinary team in the resolution of a clinician's uncertain cases [*(11 + 24)/(11 + 25)*]. Taken together with the results of Table 2a, overall accuracy of the integration method for the total study sample was 88% [(*11 + 24 + 95 + 85 + 27)/(11 + 24 + 1 + 95 + 8 + 21 + 85 + 3 + 27)*].

### Standard accuracy analysis

From the classification results of Tables 2011a–b, standard accuracy analysis can be performed for true and false results of the integration method, because the outcomes of the reference standard (multidisciplinary team) have been matched to those of the integration method (“Less likely to meet criterion E” vs. “ADHD confirmed” or “ADHD more likely to be confirmed”). However, this is not the case for the clinician alone (“uncertain” or “ADHD”). Integration method results are intended to provide a progression toward improved criterion E certainty, and are therefore expected to be somewhat different from results of the clinician alone. To allow comparison, some assumptions were then required to analyze accuracy for the clinician alone in terms of the reference standard (see footnotes of Table 3). As such, accuracy results for the integration method do represent valid estimates. However, accuracy results for the clinician alone represent assumption-based estimates that allow illustration of the potential for improvement. With the above context in mind, results support that integration of the EEG information with a clinician's ADHD evaluation could improve diagnostic accuracy from 61% to 88% (Table 3).

**Table 3:** Standard accuracy analysis with Multidisciplinary Team as reference standard, demonstrating the potential that a clinician integrating EEG could improve accuracy of differential diagnosis in a complex clinical population

	Clinician^1^	*n*	Clinician + EEG	*n*
Specificity, % (95% CI)	36 (29–44)	145	94 (89–97)	145
Sensitivity, % (95% CI)	89 (83–93)	130	82 (74–87)	130
Positive Predictive Value*, % (95% CI)	56 (49–62)	209	92 (86–96)	115
Negative Predictive Value*, % (95% CI)	79 (67–87)	66	85 (79–90)	160
Overall Accuracy, % (95% CI)	61 (55–67)	275	88 (84–91)	275

95% CI, 95% confidence interval. *Reference prevalence for positive condition: 47% (130/275).

1 Assumptions for calculation of clinician accuracy results: Positive = Multidisciplinary Team: ADHD confirmed or ADHD more likely to be confirmed. Negative = Multidisciplinary Team: negative or less likely to meet criterion E. In addition because FDA requirements did not allow exclusion of data from the analyses, clinician: uncertain was treated as “negative” for the results presented here.

### Generalizability and reliability

Results in Tables 2011 and 3 support that a diagnosis rendered by a clinician using the EEG integration method would be more likely to converge upon the diagnostic results of a multidisciplinary team (in particular toward more rigorous application of criterion E). To examine generalizability of these results, overall accuracy results of the integration method across demographics, comorbidities, and site characteristics have been shown to be consistent (see Table S1). To test reliability for the EEG biomarker, ICC was determined using TBR repeated measures (*ICC(C,1), 0.83*).

### Differences between groups

The proposed integration method is intended to help improve certainty with criterion E, as supported by results of Tables 2 and 3. One possible explanation for the apparent improvement is that patients with relatively lower TBR were also found to be more likely to have other conditions that could affect criterion E certainty (*10 significant results; P ≤ 0.05*), as shown in Tables 2000a–c.

**Table 4:** Results of χ^2^ analysis, showing in ADHD and uncertain cases (per site clinicians) with relatively lower TBR: (a) medical mimics were more likely, (b) anger and medication issues were more likely, and (c) conditions that could impact an ADHD evaluation were more likely

Condition	Clinician:ADHD or Uncertain	Clinician:ADHD or Uncertain	*P* value
	EEG (TBR level):Low to Moderate	EEG (TBR level):High	
	Clinician + EEG:Less Likely to Meet Criterion E^1^	Clinician + EEG:ADHD Confirmed/ADHD More Likely To Be Confirmed^1^	
	(*n* = 130)	(*n* = 115)	
	(% with condition)	(% with condition)	
(a)			
Medical or neurological conditions known to mimic ADHD: head injury with ongoing impairment headaches affecting attention auditory processing disorder sensory integration dysfunction substance abuse tobacco exposure influence of asthma medications neuro-maturational delays/soft signs congenital encephalopathy cerebral palsy mild mental retardation anemia	22	4	<0.001*
Uncorrected vision or hearing problems	32	20	0.029*
(b)			
Anger issues: anger as a primary concern anger arising with ADHD medications	15	4	0.007*
Aggression issues: aggression as a primary concern aggression arising with ADHD medications probable to definite conduct disorder or oppositional defiant disorder	38	26	0.052
History of no improvement with ADHD medications	8	1	0.010*
History of adverse events with ADHD medications: headaches nausea weight loss lethargy insomnia irritability withdrawal depression anxiety compulsiveness tics cardiac problems	15	5	0.010*
(c)			
Multidisciplinary team supported overall possibilities: medical mimics^2^ anger as primary concern^3^ aggression as primary concern^3^ medication issues^3^ possible exclusionary disorders: pervasive developmental disorders psychotic disorders bipolar disorders disorders caused by a stressing event: post-traumatic stress disorder adjustment disorders	51	24	<0.001*
Multidisciplinary team supported that case may need more detailed differential diagnosis	22	9	0.004*
Clinician's initial unstructured interview did not support ADHD	39	19	0.001*
Teacher rating scales were inconsistent with ADHD	27	22	0.424
Child and/or parent had record of dissatisfaction with ADHD diagnosis	12	3	0.008*
Child had record of satisfactory academic and intellectual performance^4^	13	4	0.017*

* Significant difference (*P *≤* *0.05; for correction used, see Methods: Statistical Analysis).

1 See Table 1.

2 See Table 4a for description.

3 See Table 4b for description.

4 Reported doing well academically/intellectually; no special education; no grade retention.

Results show that ADHD and uncertain cases (per site clinicians) with relatively lower TBR were significantly more likely to have complicating conditions including: (1) medical or neurological conditions that could mimic ADHD (Table 4a), (2) anger and medication issues (Table 4b), and (3) overall possibility of complicating conditions that could impact an ADHD evaluation (Table 4c). The results are consistent with the full designation for these cases from the integration method: “*less likely to meet criterion E… recommend resolution by further clinical testing for other conditions*”. These subjects are much more likely to have other conditions that could affect the clinician's decision on criterion E.

Related TBR results have been provided in the supplement (see Table S2), and show consistent significant TBR differences between cases with: (1) condition present and less likely to meet criterion E, and (2) condition absent and ADHD confirmed/more likely to be confirmed, with multidisciplinary team as reference standard, analyzed for the conditions as presented in Table 4. Further common conditions were also examined including anxiety disorder, mood disorder, disruptive disorder, and learning disorder. The results support that relatively higher TBR is associated with ADHD (in cases more likely to meet criterion E), and not associated with any of the conditions examined (see Table S2).

### TBR effect size

In Table 5, TBR results show statistical power specific to supporting certainty of criterion E per the multidisciplinary team (*Cohen's d, 1.53*). In addition, results support that the observed TBR effect size can vary based on: (1) application of TBR (criterion E certainty vs. ADHD diagnosis) and (2) reference standard (multidisciplinary team vs. individual clinician). In other words, the TBR results show sufficient statistical power to improve certainty of criterion E (per multidisciplinary team), but not to diagnose ADHD (per individual clinician).

**Table 5:** Theta/beta ratio (TBR) results, showing sufficient statistical power to improve certainty of criterion E (per Multidisciplinary Team), but not to diagnose ADHD (per individual clinician)

	TBR, Mean	Standard Deviation	*n*	*P* value	Cohen's *d*
Clinician (to diagnose ADHD)					
ADHD	4.98	2.27	209	0.052	0.38
Not ADHD	4.12	2.01	30		
Multidisciplinary Team (to improve certainty of criterion E)					
ADHD confirmed/more likely^1^	6.22	2.24	127	<0.001*	1.53
Less likely to meet criterion E	3.38	1.31	118		

* Significant difference (*P *≤* *0.05).

1 ADHD confirmed/ADHD more likely to be confirmed.

**Figure 1:**
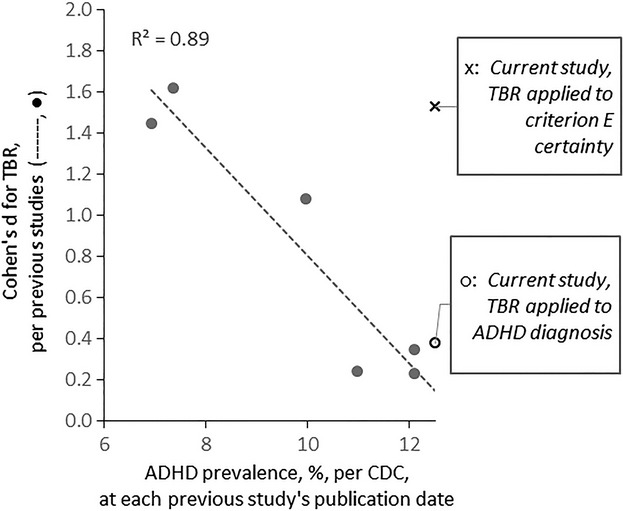
Cohen's *d* values (for each of six previous studies that were included in a recent meta-analysis by Arns et al. 2013 to represent ages 6–18 years) have been plotted relative to ADHD prevalence (per CDC estimates applied to each study's publication date (CDC, 2014; Visser et al. 2014)) (●). Linear fit (- - - -) shows an inverse relationship (*R*^*2*^*, 0.89*). In the current study, applying TBR to ADHD diagnosis per an individual clinician (o) has reduced statistical power as predicted by the trend; however, applying TBR to improve certainty of criterion E per a Multidisciplinary Team (x) restores statistical power to prior levels.

The current observation of variation in TBR effect size may provide insights into previous TBR studies. Figure1 shows that a previously observed trend of a decline over recent years in statistical power of TBR when applied to ADHD diagnosis, as reported in a recent meta-analysis (Arns et al. 2013), can be associated with a rapid increase in ADHD prevalence that occurred over the same time period, 1999–2012, as reported by the Centers for Disease Control and Prevention (CDC) (CDC 2014; Visser et al. 2014). Cohen's *d* values (for each of the six studies included in the meta-analysis for ages 6–18 years) have been plotted relative to ADHD prevalence (per CDC estimates at each study publication date) (●). Linear fit (- - - -) shows an inverse relationship (*R*^*2*^*, 0.89*). Additional inclusion of the current TBR results in the plot demonstrates support that the rapid change in ADHD prevalence may be due in part to a less stringent approach to criterion E by regular ADHD evaluations. As shown in Figure1, applying TBR to ADHD diagnosis per an individual clinician does have reduced statistical power (o) as predicted by the trend (- - - -); however, applying TBR to improve certainty of criterion E per a multidisciplinary team restores statistical power to prior levels (x).

**Figure 2:**
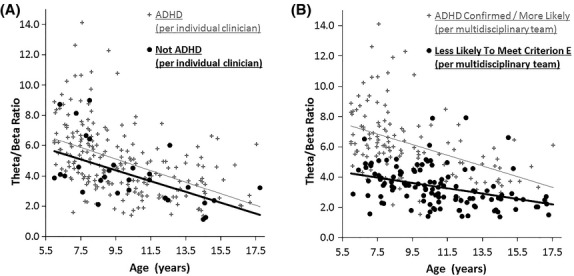
TBR of each individual subject plotted by age. As shown by slopes of linear fits and by *overlap* of groups, (A) supports only an age effect, when TBR is applied to diagnose ADHD per individual clinician (*ADHD, m: −0.38, b: 8.76, R*^*2*^*: 0.22; not ADHD, m: −0.36, b: 7.79, R*^*2*^*: 0.31*). As shown by slopes of linear fits and by *separation* of groups, (B) supports presence of both age effect and assessment power, when TBR is applied to improve certainty of criterion E per Multidisciplinary Team (*ADHD confirmed/ADHD more likely to be confirmed, m: −0.35, b: 9.37, R*^*2*^*: 0.19; Less likely to meet criterion E, m: −0.18, b: 5.47, R*^*2*^*: 0.14*).

The current observation of variation in TBR effect size may also provide insights into age effects observed in previous TBR studies. In Figure2, individual TBR results of the current study were plotted by age. Figure2A supports only an age effect, when TBR is applied to diagnose ADHD per individual clinician. This outcome is consistent with TBR application and analysis of a recent study which speculated that TBR may only be of value as an age predictor (Liechti et al. 2013). However, Figure2B supports presence of both age effect and assessment power, when TBR is applied to improve certainty of criterion E per multidisciplinary team. These current results provide further support that TBR findings between studies may vary based on TBR application and reference standard.

### Missing data analysis

Missing data analysis was conducted to evaluate the 32 subjects who did not have “complete” EEG recordings, by which “complete” refers to a quality standard set prior to study initiation requiring at least 15 epochs (30 sec) with minimal to no artifact. One-way ANOVA analysis (significance level, 0.05) showed the 32 subjects with incomplete EEG recordings were younger than those with complete EEG recordings (mean age of 8.6 vs. 10.1 years; *P *=* *0.006), had higher CGI-S severity scores (mean score of 4.6 vs. 3.7; *P *<* *0.001) and lower CGAS functioning scores (mean score of 50.8 vs. 55.7; *P *=* *0.003). Of the 32 excluded data sets, 29 had between 1 and 14 epochs. Further analysis with inclusion of data sets with 1–14 epochs was shown to have minimal effect on accuracy results. Accuracy was 86% (95%CI: 81–89; *n*: 304) with missing data included, compared to 88% (95%CI: 84–91; *n*: 275) observed per planned study analyses.

## Discussion

### Summary

The proposed assessment aid involves a method to integrate an EEG biomarker (TBR) with a clinician's regular ADHD evaluation. To evaluate the assessment aid, we conducted a prospective, triple-blinded, multi-site, clinical cohort study with a reference standard based on an independent multidisciplinary team. Results support that the EEG-based assessment aid may help improve accuracy of ADHD diagnosis by supporting greater criterion E certainty. Of 209 patients meeting ADHD criteria per a site clinician's judgment, 93 were separately found by the multidisciplinary team to be less likely to meet criterion E, implying possible overdiagnosis by clinicians in 34% of the total clinical sample (*93/275*). Of those 93, 91% were also identified by EEG, showing a relatively lower TBR (*85/93*) (Table 2a). Further, the integration method was in 97% agreement with the multidisciplinary team in the resolution of a clinician's uncertain cases (*35/36*) (Table 2b). TBR showed statistical power specific to supporting certainty of criterion E per the multidisciplinary team (*Cohen's d, 1.53*) (Table 5 and Figs.2). Patients with relatively lower TBR were more likely to have other conditions that could affect criterion E certainty (*10 significant results; P ≤ 0.05*) (Table 4). Integration of this information with a clinician's ADHD evaluation could help to improve diagnostic accuracy from 61% to 88% (Table 3).

### Comparison with previous EEG research

The current approach for integrating an EEG biomarker with a clinician's ADHD evaluation represents a divergence from methods used in previous EEG studies. Prior studies examined methods that directly identify ADHD. In contrast, the current method is intended to identify cases with ADHD symptoms that are either more or less likely to meet criterion E. In other words, prior studies used EEG variable cutoffs that were optimized to differentiate ADHD patients from controls (Monastra et al. 2001; Barry et al. 2003; Snyder and Hall 2006; Arns et al. 2013). In contrast, the work presented here examined cutoffs designed with the objective of parsing suspected ADHD patients into test-result subgroups with clinically meaningful differences that are relevant to criterion E certainty, as well as to the need for more extended medical and neuropsychiatric evaluation. Because the current cutoffs were developed and documented prior to uncovering the blind in this study (using development data separate from that of the current study; see Methods: EEG Collection and Analysis), the current results provide independent and blinded validation of our proposed method for integration of an EEG biomarker into the clinical setting.

In the current study, TBR showed sufficient statistical power to improve certainty of criterion E (per multidisciplinary team), but not to diagnose ADHD (per individual clinician) (Table 5). This finding may offer some insights toward results of previous studies. One prior study which speculated that TBR may only be of value as an age predictor, used TBR applied to ADHD diagnosis, rather than the current approach of TBR applied to criterion E certainty (Liechti et al. 2013). In the current study when TBR is applied to diagnose ADHD per individual clinician, only an age effect is observed (Fig.2A), however, when TBR is applied to address certainty of criterion E per multidisciplinary team, there is presence of both an age effect and assessment power (Fig.2B). One implication is that the observed TBR effect size can vary based on: (1) application of TBR (criterion E certainty vs. ADHD diagnosis) and (2) reference standard (multidisciplinary team vs. individual clinician).

The current observation of variation in TBR effect size may also provide insights into a recent meta-analysis which observed a trend of decline over recent years in statistical power of TBR applied to ADHD diagnosis (Arns et al. 2013). Figure1 shows that decline of TBR effect size in recent studies can be associated with a rapid increase in CDC-reported ADHD prevalence (CDC 2014; Visser et al. 2014) that occurred over the same time period, 1999–2012 (*R*^*2*^*, 0.89*). Per the current study, applying TBR to ADHD diagnosis per an individual clinician does have reduced statistical power as predicted by the trend. However, applying TBR to improve certainty of criterion E per a multidisciplinary team restores statistical power to prior levels. One possible implication is that the rapid change in ADHD prevalence from 1999 to 2012 may be due in part to a more inclusive approach to ADHD by regular evaluations (i.e., a less stringent approach to criterion E).

The possibility of an increase in ADHD prevalence due to less stringent criterion E has been supported by epidemiological and multidisciplinary studies. Epidemiological research has shown that ADHD prevalence is reduced after application of more rigorous diagnostic criteria including focus on other conditions that could account for ADHD symptoms (Rohde et al. 1999; Polanczyk and Jensen 2008). Along the same lines, previous clinical findings have shown that when a multidisciplinary model is applied, a significant number of children and adolescents presenting with ADHD-like concerns may be determined as having other primary diagnoses (Pearl et al. 2001, 2015). The current study showed similar findings, namely that out of 209 patients meeting ADHD criteria per a site clinician's judgment, 93 were separately found by the multidisciplinary team to be less likely to meet criterion E, implying possible overdiagnosis by clinicians in 34% of the total clinical sample (*93/275*) (Table 2a).

The current finding of a potential 34% overdiagnosis rate by regular ADHD evaluations is consistent with findings of previous studies which found 14–38% overdiagnosis (Elder 2010; Chilakamarri et al. 2011; Bruchmuller et al. 2012). For instance, one recent study showed that ADHD was overdiagnosed in 38% of patients with depression and 29% of patients with bipolar disorder (Chilakamarri et al. 2011). Another study showed 14% overdiagnosis of ADHD when only anxiety was present (Bruchmuller et al. 2012). Studies have also shown that misdiagnosis of ADHD is more common in children who are relatively young for their school grade, accounting for as much as 20% of ADHD cases (Elder 2010; Morrow et al. 2012). These studies imply that the rapid increase in ADHD prevalence per CDC report may be due at least in part to a less stringent approach to criterion E.

On a related note to EEG, of the 93 cases in the current study meeting ADHD criteria per an individual clinician's judgment but less likely to meet criterion E per the multidisciplinary team, 91% were also identified by a relatively lower TBR (Table 2a). This outcome implies that the proposed integration method could be used to help address overdiagnosis by prompting toward a more stringent application of criterion E in the appropriate cases. Table 4 shows that ADHD and uncertain cases (per site clinicians) with relatively lower TBR were significantly more likely to have other conditions that could affect criterion E certainty, including: (1) medical or neurological conditions that could mimic ADHD, (2) anger and medication issues, and (3) overall possibility of complicating conditions that could impact an ADHD evaluation. These findings are consistent with the full designation for these cases from the integration method: “*less likely to meet criterion E… recommend resolution by further clinical testing for other conditions*”.

### Comparison with other related research

In the current study, the test-result subgroups with designations “ADHD confirmed/ADHD more likely to be confirmed” are characterized by a relative increase in TBR (Table 5), which may be consistent with findings in neuroimaging studies. For example, neuroimaging studies reported reduced blood flow, reduced metabolic activity, differences in activation, and decreased frontal lobe volume in the frontal regions of many ADHD subjects (Lou et al. 1984; Zametkin et al. 1990; Sowell et al. 2003; Bush et al. 2005; Dickstein et al. 2006; Cortese 2012). Because EEG is limited in spatial resolution, future studies might use neuroimaging methods to compare neurological substrates in test-result subgroups per the proposed integration method.

Our medication response findings are consistent with results of previous studies. Results showed that ADHD and uncertain cases (per site clinicians) with relatively lower TBR were more likely to have had a history of nonresponse and adverse events on ADHD medications (Table 4b). Previous EEG studies have also found that ADHD patients with relatively lower TBR values were less likely to respond favorably to stimulants (Clarke et al. 2002b,c). Neuroimaging studies have indicated that treatment with stimulants normalized hypoperfusion and under-activation associated with ADHD, particularly in the frontal region (Lou et al. 1984; Lou and Hendrickson 1989; Rubia et al. 2011a,b). Similarly, treatment with stimulants normalized EEG theta and beta abnormalities in subjects with ADHD (Clarke et al. 2002a, 2003). Taken together, these results suggest a possible association between frontal cortical dysfunction (demonstrated by EEG and neuroimaging results) and a positive response to stimulants in patients with ADHD.

The current results are consistent with previous studies recognizing differences between neuropsychological test-result subgroups of patients with ADHD. Neuropsychological testing of executive functions has been reported to separate patients with ADHD into two subgroups differing in academic outcomes: grade retention and academic achievement (Nigg et al. 2004). Likewise in the current study, EEG testing separated patients with ADHD into subgroups with comparable differences related to academics (grade retention and academic achievement, as well as special education requirement; Table 4c).

Future validation studies of a refined ADHD phenotype based upon biomarkers may include neuropsychological testing, genomic measures, or differential response to treatment, and lead to more objective diagnostic procedures and more personalized ADHD treatment. One caveat is that such studies may need to implement methods to account for the potential effect of a CDC-reported rapid increase in ADHD prevalence. The increase in ADHD prevalence has been significant, at 3% per year from 1997 to 2006, and then 5% per year from 2003 to 2011 (CDC 2014; Visser et al. 2014). As an example of a potential effect, the current study showed that a previously observed trend of a decline over recent years in statistical power of TBR when applied to ADHD diagnosis, as reported in a recent meta-analysis (Arns et al. 2013), may be due in part to the rapid increase in ADHD prevalence, possibly related to a less stringent application of criterion E (see Fig.1 and Discussion: Comparison with Previous EEG Research). As a further example, TBR assessment power in Figure2 was only apparent with application of a more rigorous reference standard focused on criterion E (Fig.2B).

Along the same lines, the current study's observation that TBR effect size varies relative to the reference standard may imply that ADHD heterogeneity noted in biomarker studies may be due in part to clinical diagnostic variation (Figs.2; Tables 2000 and 2013). For example, a recent study observed in groups diagnosed with ADHD and comorbid depression that mean TBR was reduced (Loo et al. 2013). Similarly in our study, accuracy of clinician's ADHD evaluation + EEG was reduced from 88% (95%CI: 84–91%) to 81% with presence of a mood disorder (see Table S1). Loo et al. also observed in groups diagnosed with ADHD and comorbid disruptive behavior disorder that mean TBR was increased. Likewise in our study, accuracy of clinician's ADHD evaluation + EEG increased to 97% with presence of another disruptive behavior disorder. The comorbid trends shown by Loo et al. and by the current study may be due to mediating effects on TBR as interpreted by Loo et al., however, diagnostic variation in the clinical evaluation cannot be ruled out. A recent study showed that ADHD was overdiagnosed in 38% of patients with depression and 29% of patients with bipolar disorder (Chilakamarri et al. 2011), and ADHD overdiagnosis could simulate a comorbid mediating effect in neurobiological outcomes. A more inclusive approach to ADHD evaluation could lead to a reduction of TBR effect size, as shown in Figure1 and Table 5. Irrespective of the interpretation of causality, both study outcomes do provide support that comorbid mood and disruptive disorders may be potential sources of heterogeneity. Future research might further examine diagnostic variation vs. heterogeneity by implementing a longitudinal design, including monitoring of stability of the diagnosis (and treatment response when applicable).

Another reported source of heterogeneity may be neurobiological differences between boys and girls. Studies from Dupuy et al. 2011, 2013 showed sex differences in comprehensive EEG profiles of ADHD DSM-IV-TR subtypes. However, in the current approach with a single EEG variable integrated with a clinician's ADHD evaluation, there was no sex difference in accuracy, with boys and girls each at 88% (see Table S1). Whereas Dupuy's analysis of multiple EEG variables (eyes closed) supported determination of differences between boys and girls of different ADHD subtypes, it appears that in the current approach, the single variable, TBR (eyes open), highlighted sufficient sex similarity to support 88% accuracy with the integration model. Another possible explanation for our consistent accuracy between boys and girls may be that the application of EEG in a clinical integration model allowed for accounting of sex differences by the clinician.

## Clinical implications

A risk/benefit analysis underscores the main clinical implication of the proposed approach, which is that integration of the biomarker may increase specificity in ADHD diagnosis (Table 3). This outcome may be explained by EEG-based subgroup differences presented in Tables 4a–c, which show that the EEG approach could inform a clinician's application of criterion E, which would lead to an increase in specificity. Specificity improvement can be traced through the classification results (Tables 2a–b), as follows. Of the 209 patients meeting ADHD criteria per a site clinician's judgment, 93 were separately found by the multidisciplinary team to be less likely to meet criterion E, implying possible overdiagnosis by clinicians in 34% of the total clinical sample (*93/275*). Of those 93, 91% were also identified by EEG, showing a relatively lower TBR (*85/93*). Further, the integration method was in 97% agreement with the multidisciplinary team in the resolution of a clinician's uncertain cases (*35/36*).

The accompanying risk is that some patients may be delayed before receiving ADHD treatment while they are receiving unnecessary further testing due to incorrect recommendations by the integration method. Of the 116 ADHD cases confirmed by the multidisciplinary team, 21 were identified by the integration method as less likely to meet criterion E, implying an unnecessary prompt for further clinical testing by the integration method in 8% of the total clinical sample (*21/275*). In addition, it is important to note that in the cases in which ADHD is determined to be less likely to be the primary diagnosis, it may still be necessary to treat ADHD-related symptoms, in particular in the cases in which these symptoms have not been resolved after successful treatment and management of the primary condition.

## Limitations

The current study's sampling was limited to subjects who could sit still for at least 30 sec of EEG recording. Missing data analysis showed that younger subjects with more severe symptoms and lower functioning were less likely to receive a complete EEG recording, by which “complete” refers to a quality standard set prior to study initiation requiring at least 15 epochs (30 sec) with minimal to no artifact. Further analysis with inclusion of missing data (i.e., data sets with 1–14 epochs) was shown to have minimal effect on accuracy results (see Results: Missing Data Analysis). Therefore, implementation of the proposed EEG biomarker with these subjects is viable, but rate of acquiring acceptable epochs needs to be improved. The main cause of epoch rejection in younger children with more severe symptoms is motion artifact, which can be reduced by modifications including: (1) use of standard calming methods with children during EEG collection, and (2) use of electrode setup that reduces movement of individual electrodes by application of elastic band, cap, or tape.

The report of current results includes comparisons with previous EEG studies, however, the comparisons may have limitations due to possible differences in EEG materials and methods between studies. In the standardization of the current study's EEG materials and methods, areas of possible variation were examined and optimized specifically to support consistent TBR determination. Areas that were standardized included: artifacting methods (e.g., noise tracking and recognition, rules for noise inclusion/exclusion, selection of electrodes to guide artifacting, use of electro-oculography, use of a voltage cutoff, level selected for voltage cutoff), device characteristics (e.g., frequency response of the device, electrode materials), TBR analysis (e.g., reference, filters, calculation method for TBR, frequency ranges for theta and beta, TBR cutoffs designed per the intended use, handling of EEG epochs during processing), and other EEG collection factors in the study protocol (e.g., recommendations to subject prior to collection visit, medication washout including types and duration, tracking of factors that could affect EEG such as sleep and alertness, minimized duration of set up to limit subject fatigue (5 min), minimized visit requirements prior to EEG collection to limit fatigue, impedance range allowed, accounting for electromagnetic interference in the recording area including computer power supply and local interference). Also of note is the implementation of a blinding protocol between EEG methods and ADHD evaluation. Because of the potential for variation as evaluated in the standardization process, it cannot be ruled out that differences of EEG materials and methods between studies may have contributed to differences observed in results between studies.

## Conclusions

By meeting De Novo requirements of US Food and Drug Administration as well as Class I evidence requirements of American Academy of Neurology and American Clinical Neurophysiology Society, the current assessment aid has been held to higher validation standards than commonly used assessment aids. In addition, the current assessment aid is novel in providing a validated method for integration into clinical practice and addressing an important issue in ADHD evaluation, namely sufficient determination of criterion E.

Study results showed that in patients meeting ADHD criteria per an individual clinician, those with a relatively lower theta/beta ratio were more likely to have conditions that may account for ADHD symptoms or otherwise impact the ADHD evaluation (*10 significant results; P ≤ 0.05*). And, ADHD cases with higher theta/beta ratio were less likely to have those conditions. Integration of this information with a clinician's regular ADHD evaluation could improve diagnostic accuracy from 61% to 88%, in particular when addressing criterion E in a complex clinical population in which all patients have ADHD-like symptoms but not all have ADHD. Results support that the EEG-based assessment aid may help the clinician to determine whether symptoms are better explained by another condition.

Application of EEG in a clinical integration model produced results that are consistent with those of prior studies of neuroimaging, neuropsychology, and medication effects. The current results also support that a source of heterogeneity in ADHD research may be diagnostic variation in the clinical evaluation. Future validation studies of a refined ADHD phenotype based upon biomarkers may include neuropsychological testing, genomic measures, or differential response to treatment, and lead to more objective diagnostic procedures and more personalized ADHD treatment.

## Supporting Information

**Table S1.** Accuracy analysis with Multidisciplinary Team as reference standard, showing clinician integrating EEG would have consistent accuracy across various categories. **Table S2.** Theta/beta ratio (TBR) results, with comparison of: (1) condition present and less likely to meet criterion E versus (2) condition absent and ADHD confirmed/more likely to be confirmed, per Multidisciplinary Team as reference standard.
