# Supplementary material for: Integration of an EEG biomarker with a clinician's ADHD evaluation
Source: Brain Behav. 2015 Mar 5;5(4):e00330. doi: 10.1002/brb3.330 (PMC4356845; doi:10.1002/brb3.330)
Supplement: Supplementary file 1 — Table S1. Accuracy analysis with Multidisciplinary Team as reference standard, showing clinician integrating EEG would have consistent accuracy across various categories. Table S2. Theta/beta ratio (TBR) results, with comparison of: (1) condition present and less likely to meet criterion E versus (2) condition absent and ADHD confirmed/more likely to be confirmed, per Multidisciplinary Team as reference standard. [file brb30005-e00330-sd1.docx]

**Supporting Information**

**Table S1.** Accuracy analysis with Multidisciplinary Team as reference standard, showing clinician integrating EEG would have consistent accuracy across various categories.

| Category | Subgroup | *n* | Clinician + EEG  Accuracy, % |
| --- | --- | --- | --- |
| Age | 6-11 years | 201 | 88 |
| Age | 12-17 years | 74 | 88 |
| Sex | Female | 97 | 88 |
| Sex | Male | 176 | 88 |
| Race | White | 195 | 90 |
| Race | Non-white | 72 | 85 |
| Socioeconomic status | Upper/middle | 136 | 90 |
| Socioeconomic status | Working/Lower | 139 | 86 |
| An Anxiety Disorder | present | 41 | 88 |
| An Anxiety Disorder | absent | 234 | 88 |
| A Mood Disorder | present | 27 | 81* |
| A Mood Disorder | absent | 248 | 89 |
| A Disruptive Disorder | present | 67 | 97* |
| A Disruptive Disorder | absent | 208 | 85 |
| A Learning Disorder | present | 85 | 86 |
| A Learning Disorder | absent | 190 | 89 |
| Site Type | Psychiatry | 101 | 85 |
| Site Type | Pediatrics | 96 | 86 |
| Site Type | Psychology | 78 | 94* |
| Site Sector | Academic | 105 | 90 |
| Site Sector | Private | 170 | 87 |

*Outside of 95% CI (84-91%) for accuracy in total sample (see Table 3)

**Table S2.** Theta/beta ratio (TBR) results, with comparison of: 1) condition present & less likely to meet criterion E vs. 2) condition absent & ADHD confirmed / more likely to be confirmed, per Multidisciplinary Team as reference standard.

|  | Condition is present  and  Less likely to meet criterion E | | | Condition is absent  and  ADHD confirmed / more likely^1^ | | |  |
| --- | --- | --- | --- | --- | --- | --- | --- |
| Condition | TBR, Mean | SD | *n* | TBR, Mean | SD | *n* | *P* value |
| An Anxiety Disorder (*Table S1*) | 3.36 | 1.68 | 17 | 6.20 | 2.29 | 115 | <0.001* |
| A Mood Disorder (*Table S1*) | 3.38 | 1.43 | 16 | 6.31 | 2.22 | 122 | <0.001* |
| A Disruptive Disorder (*Table S1*) | 3.20 | 1.30 | 39 | 6.09 | 2.29 | 110 | <0.001* |
| A Learning Disorder (*Table S1*) | 3.10 | 1.03 | 30 | 6.12 | 2.32 | 81 | <0.001* |
| A Medical Mimic (*Table 4a*) | 3.15 | 1.28 | 30 | 6.21 | 2.22 | 123 | <0.001* |
| An Uncorrected Vision or Hearing Problem (*Table 4a*) | 3.27 | 1.05 | 44 | 6.04 | 2.35 | 106 | <0.001* |
| Anger Issues (*Table 4b*) | 2.85 | 0.76 | 17 | 6.24 | 2.28 | 122 | <0.001* |
| Aggression Issues (*Table 4b*) | 3.28 | 1.28 | 52 | 6.07 | 2.28 | 100 | <0.001* |
| History of no improvement with ADHD medications (*Table 4b*) | 3.30 | 1.06 | 10 | 6.22 | 2.25 | 126 | <0.001* |
| History of adverse events with ADHD medications (*Table 4b*) | 3.26 | 0.92 | 20 | 6.20 | 2.22 | 121 | <0.001* |
| Multidisciplinary Team – overall possibilities of complicating conditions (*Table 4c*) | 3.22 | 1.19 | 68 | 6.08 | 2.12 | 102 | <0.001* |
| Multidisciplinary Team – supported need for more detailed differential diagnosis (*Table 4c*) | 3.10 | 0.94 | 30 | 6.15 | 2.16 | 118 | <0.001* |
| Clinician’s initial unstructured interview did not support ADHD. (*Table 4c*) | 2.94 | 1.03 | 45 | 6.23 | 2.27 | 97 | <0.001* |
| Teacher rating scales were inconsistent with ADHD. (*Table 4c*) | 3.38 | 1.63 | 39 | 6.12 | 2.29 | 106 | <0.001* |
| Child and/or parent had record of dissatisfaction with ADHD diagnosis. (*Table 4c*) | 2.88 | 0.88 | 15 | 6.25 | 2.26 | 124 | <0.001* |
| Child had record of satisfactory academic and intellectual performance. (*Table 4c*) | 3.49 | 0.83 | 15 | 6.18 | 2.27 | 120 | <0.001* |

SD = standard deviation. *Significant difference (*P* ≤ 0.05). ^1^ADHD confirmed / ADHD more likely to be confirmed.
